# Right ventricular dysfunction in patients with Brugada-like electrocardiography: a two dimensional strain imaging study

**DOI:** 10.1186/1476-7120-9-30

**Published:** 2011-11-16

**Authors:** Kazuya Murata, Takeshi Ueyama, Takeo Tanaka, Yoshio Nose, Yasuaki Wada, Masunori Matsuzaki

**Affiliations:** 1 Division of Laboratory, Yamaguchi University Hospital, Ube, Japan; 2 Department of Medicine and Clinical Science, Yamaguchi University Graduate School of Medicine, Ube, Japan

**Keywords:** Brugada-like ECG, speckle tracking, two-dimensional strain imaging, TEI index, sodium channel blocker

## Abstract

**Background:**

Sodium channel blockers augment ST-segment elevation in the right precordial leads in patients undergoing Brugada-type electrocardiography (ECG). However, their effect on echocardiographic features is not known. We address this by assessing global and regional ventricular function using conventional Doppler and two- dimensional (2D) speckle tracking techniques.

**Methods:**

Thirty-one patients with Brugada-type ECG were studied. A pure sodium channel blocker, pilsicainide, was used to provoke an ECG response. The percentage longitudinal systolic myocardial strain at the base of both the right ventricular (RV) free wall and the interventricular septum wall was measured using 2D speckle tracking. Left ventricular (LV) and RV myocardial performance (TEI) indices were also measured.

**Results:**

The pilsicainide challenge provoked a positive ECG response in 13 patients (inducible group). In the inducible group, longitudinal strain was significantly reduced only at the RV (-27.3 ± 5.4% vs -22.1 ± 3.6%, *P *< 0.01), and both RV and LV TEI indices increased (RV: 0.19 ± 0.09 vs 0.27 ± 0.11, *P *< 0.05; LV: 0.30 ± 0.10 vs 0.45 ± 0.10, *P *< 0.01) after pilsicainide administration.

**Conclusions:**

Temporal and spatial analysis using the TEI index and 2D strain imaging revealed the deterioration of global ventricular function associated with conduction disturbance and RV regional function in patients with Brugada-type ECG and coved type ST elevation due to administration of a sodium channel blocker.

## Background

Brugada syndrome is characterized by ST-segment elevation in right precordial leads and electrolyte disturbances associated with a high risk of cardiac sudden death [1]. Despite these ST-segment changes in the ECG in this syndrome, no structural abnormalities have been detected by conventional echocardiography. Drug challenge tests using sodium channel blockers exaggerate ST segment elevation when such elevation is initially absent, and have generally become accepted as a useful method for diagnosing Brugada syndrome [1-5]. A previous study demonstrated that a sodium channel blocker, flecainide, induced a delay in right ventricular (RV) activation in patients with Brugada syndrome, using tissue Doppler imaging (TDI) [6]. Other studies using electron-beam computed tomography scan revealed that wall motion abnormalities in the RV outflow wall were exacerbated by sodium channel blockers in patients with Brugada syndrome [3,7]. These studies specifically evaluated the timing and magnitude of RV contraction, however, the behavior of both global and regional RV and left ventricular (LV) function in patients who showed characteristic ECG changes after sodium channel blockade is unknown.

Recently, two-dimensional (2D) strain imaging, based on a speckle tracking technique, has been introduced to evaluate regional myocardial function without angle dependency [8]. Moreover, a myocardial performance index (the TEI index) derived by temporal analysis of the Doppler in- and outflow of the ventricle has been accepted as a global ventricular functional index affected by ventricular activation [9].

We aim to evaluate the echocardiographic characteristics in patients with Brugada-like ECG using 2D strain imaging and the TEI index, and to clarify whether or not changes in these indices occur according to the ECG response to a sodium channel blocker.

## Methods

### Study group

We studied 31 patients with normal LV function evaluated by 2D echocardiography with no evidence of typical Brugada signs on their baseline ECG during hospitalization. We enrolled the symptomatic patients who had a history of documented arrhythmias other than ventricular tachycardia or ventricular fibrillation or episodes related to syncope or fainting, but no documented arrhythmias at that time, and the asymptomatic patients with documented ECG abnormalities suspected of being Brugada-type or like Brugada-type abnormalities. Their ECG was either type 2, i.e. showing ST-segment elevation (saddleback type) displaying J wave amplitude or ST-segment elevation of ≥ 2 mm in more than one right precordial lead under baseline conditions, or type 3, i.e. showing right precordial ST-segment elevation of < 1 mm of the saddleback type, according to the Heart Rhythm Society and the European Heart Rhythm Association [1].

### Pilsicainide challenge

Pilsicainide was infused intravenously (1 mg/kg in 10 minutes) to provoke an ECG response. Drug administration was immediately stopped when ST elevation (> 0.5 mV), extensive QRS prolongation, unfavorable symptoms and frequent ventricular arrhythmias were observed. No complications resulted from these investigations. A positive ECG response was defined as a > 200 mcV elevation of the J wave amplitude in more than one right precordial lead [1,2] after pilsicainide infusion. Patients were subdivided into two groups according to their ECG response to pilsicainide:

Group 1: non-inducible (n = 18); pilsicainide challenge did not provoke ECG changes

Group 2: inducible (n = 13); pilsicainide challenge provoked coved type ST elevation.

Patients with atrial fibrillation, a history of significant coronary heart disease, valvular heart disease, cardiomyopathy, a history of cardiac operation, or abnormal LV systolic function were excluded from the study. The protocol was approved by the Institutional Clinical Research and Ethics Committee of the Yamaguchi University Hospital. Written informed consent was obtained from all participants before the study.

### Echocardiography and Doppler echocardiography

Echocardiography was performed with the patient in the left lateral decubitus position at end-expiration using a GE-Vingmed Vivid 7 system (GE-Vingmed Ultrasound AS, Horten, Norway) equipped with a 2-4 MHz sector transducer. Images were recorded at baseline conditions and after pilsicainide. LV end-diastolic and end-systolic dimensions were measured in the parasternal long-axis view. The LV ejection fraction was obtained using the biplane Simpson method from the apical four- and two-chamber views. Both LV and RV inflow velocities were obtained from the four- chamber view with the pulsed wave Doppler sample volume positioned at the tips of the mitral and tricuspid leaflets during diastole. The LV outflow velocity was obtained from the LV apical long-axis view with the Doppler sample volume placed below the aortic valve. The RV outflow velocity was obtained from the parasternal short-axis view at the aortic valve level with the Doppler sample volume placed below the pulmonic valve. These velocity patterns were digitized and stored in a hard disk on the ultrasound machine for 5 cardiac cycles for further analysis. Both LV and RV TEI indexes, as *(a-b)/b*, where *a *is the interval between cessation and onset of transmitral (or transtricuspid) flow and *b *is the aortic (or pulmonary) flow ejection time, were also measured by pulsed-Doppler echocardiography. The peak velocities of early (E) and late (A) mitral inflow and the deceleration time of the E wave (DcT) were measured using pulse-wave Doppler. The isovolumic contraction time (ICT) was measured as the time interval from the cessation of LV inflow to the onset of LV outflow velocity.

### 2D strain imaging

**Figure 1:**
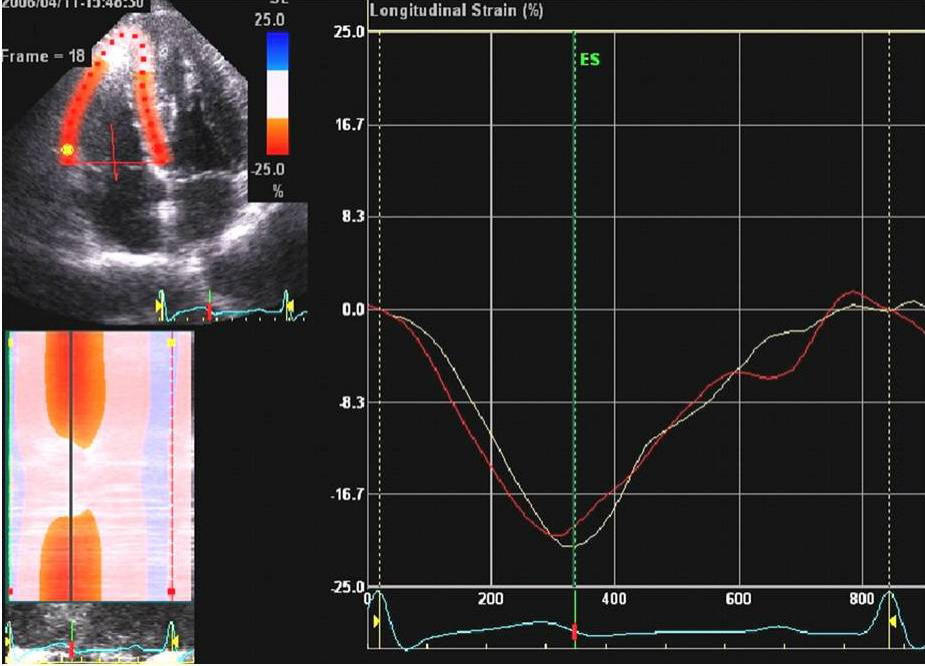
**Longitudinal myocardial strain using 2D strain imaging at the base of the right ventricular (RV) free wall (yellow) and the interventricular septum (IVS) (red)**.

LV apical four-chamber views were acquired using second harmonic gray scale imaging. Three consecutive cardiac cycles were acquired at end-expiratory apnea, and digitally stored in a hard disk for offline analysis. The images were processed on a workstation using EchoPAC Q (General Electric, Waukesha, Wis) analysis software. The 2D strain echocardiography used serial gray -scale sector images based on frame-by- frame tracking of small rectangular image blocks with stable speckle patterns [10]. The geometric position of each marker changes from frame to frame in accordance with the surrounding tissue motion. The algorithm allows tracking of the new marker location on each sequential image using correlation criteria and the sum of absolute differences. The 2D strain imaging was obtained by tracing the end-diastolic frame, and then the software automatically tracked the contour on subsequent frames. We obtained the percentage longitudinal myocardial strain using 2D strain imaging at the base of both the RV free wall and the interventricular septum (IVS) (Figure 1).

### Statistical analysis

Values were expressed as means ± SD. Data were analyzed using analysis of variance to compare group differences followed by Scheffe's multiple comparison test. Group differences after pilsicainide were analyzed using the 2-tailed Student's *t *test. *P *values less than 0.05 were considered significant.

A total of 15 studies were randomly selected for the reanalysis of 2D strain data. The measurements were repeated after 1week by same echocardiographer to assess intraobserver reproducibility. Interobserver reproducibility was assessed by a second observer who was blind to the first observer's measurements. Intraobserver and interobserver reproducibility were analyzed using both Pearson's bivariate two-tailed correlations and Bland-Altman analysis (with 95% agreement limits).

## Results

The baseline characteristics are summarized in Table 1. There were no differences in LV end-diastolic dimension, ejection fraction, and LA dimension between the two groups. Table 2 lists the electrocardiographic and echocardiographic variables in the baseline condition and after pilsicainide infusion. There were no differences in HR before and after pilsicainide in the non-inducible and inducible groups. The PR and QRS intervals and the QTc durations were significantly prolonged in both the non-inducible and inducible groups after pilsicainide infusion. The prolongation of the PR interval after pilsicainide was greater in the inducible group than that in the non-inducible group. The ratios of early to late transmitral inflow velocities (E/A) after pilsicainide were higher in both groups compared with those in the baseline condition, and the DcT was shortened in the non-inducible group after pilsicainide. Neither the RV nor the LV ejection time changed between before and after pilsicainide infusion in the non-inducible and inducible groups. Both RV and LV ICT in the inducible group and LV ICT in the non-inducible group were prolonged after pilsicainide infusion.

**Table 1:** Baseline characteristics of patients in the non-inducible, inducible and baseline positive groups

	Non-inducible	Inducible	p value
**Age (yo)**	46.9 ± 18.6	52.7 ± 16.5	NS
**Male/Female**	15/3	13/0	NS
**LVDd (mm)**	45.6 ± 3.8	46.5 ± 2.7	NS
**LVEF (%)**	68 ± 4.7	72.9 ± 4.6	NS
**LAD (mm)**	33 ± 4.4	33 ± 5.1	NS

Values are expressed as means ± SD. LVDd = left ventricular end-diastolic dimension; LVEF = left ventricular ejection fraction; LAD = left atrial dimension; NS = not significant

**Table 2:** Electrocardiographic and echocardiographic variables at baseline and after pilsicainide infusion

	Non-inducible			Inducible		
				
	Baseline	Pilsicainide		Baseline	Pilsicainide	
**HR (bpm)**	64.2 ± 8.2	66.9 ± 6.9		66.0 ± 14.1	67.7 ± 10.5	
**PR (ms)**	157 ± 20.6	183 ± 44.4	**	162 ± 14.9	211.6 ± 24.7	** ✝
**QRS (ms)**	96.5 ± 12.7	121.2 ± 12.8	**	106.4 ± 8.4	129.1 ± 11	**
**QTc (ms)**	399.2 ± 18.6	426 ± 21.5	**	401.6 ± 17.2	429.9 ± 13.9	**
**E/A**	1.33 ± 0.53	1.71 ± 0.54	**	1.56 ± 0.6	2.02 ± 0.91	**
**DcT (ms)**	205.0 ± 37.7	177.9 ± 29.9	**	187.6 ± 28.4	172.6 ± 29.2	
**RV ET (ms)**	315.3 ± 26.6	322.9 ± 28.6		326.8 ± 30.9	319.2 ± 28.0	
**LV ET (ms)**	298.6 ± 20.7	301.5 ± 23.1		304.5 ± 28.6	289.4 ± 17.8	
**RV ICT (ms)**	73.8 ± 16.5	76.1 ± 19.2		71.5 ± 6.6	89.35 ± 13.4	** ✝
**LV ICT (ms)**	75.0 ± 21.2	84.9 ± 26.5	*	62.6 ± 5.8	72.9 ± 11.6	**
**RV TEI index**	0.19 ± 0.06	0.19 ± 0.05		0.19 ± 0.09	0.27 ± 0.11	* ✝
**LV TEI index**	0.31 ± 0.08	0.34 ± 0.08		0.30 ± 0.10	0.45 ± 0.10	** ✝✝

Values are expressed as means ± SD. *P < .05 and **P < .01 *vs *corresponding value at baseline.

✝P < .05 and ✝✝P < .01 vs corresponding value in Non-inducible. *HR*, heart rate; *E/A*, ratio of early to late transmitral inflow velocity; *DcT*, deceleration time of early transmitral inflow velocity; *ET*, ejection time; *ICT*, isovolumic contraction time

### TEI index

Both RV- and LV-TEI indices at baseline were similar in the 2 groups. After pilsicainide infusion, both RV- and LV- TEI indices increased in the inducible groups. However, in the non-inducible group, the RV- and LV-TEI indices did not change.

### Longitudinal strain using 2D strain imaging

**Figure 2:**
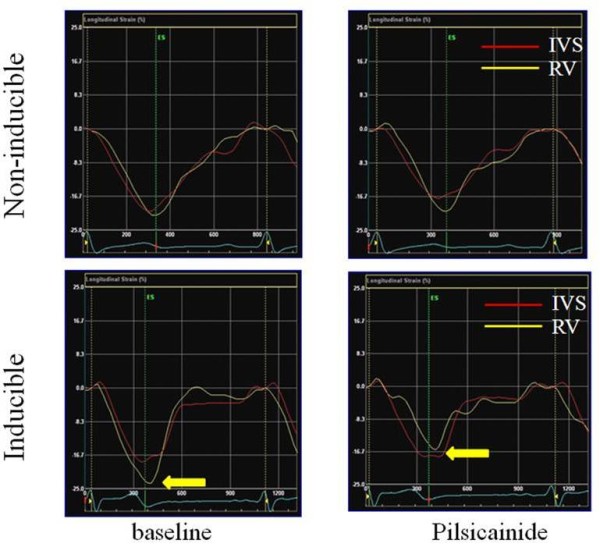
**Representative changes in longitudinal strain before and after pilsicainide infusion in the inducible and non-inducible groups**. Longitudinal strain at the base of the RV in the inducible group was significantly reduced after pilsicainide infusion (yellow arrow).

**Figure 3:**
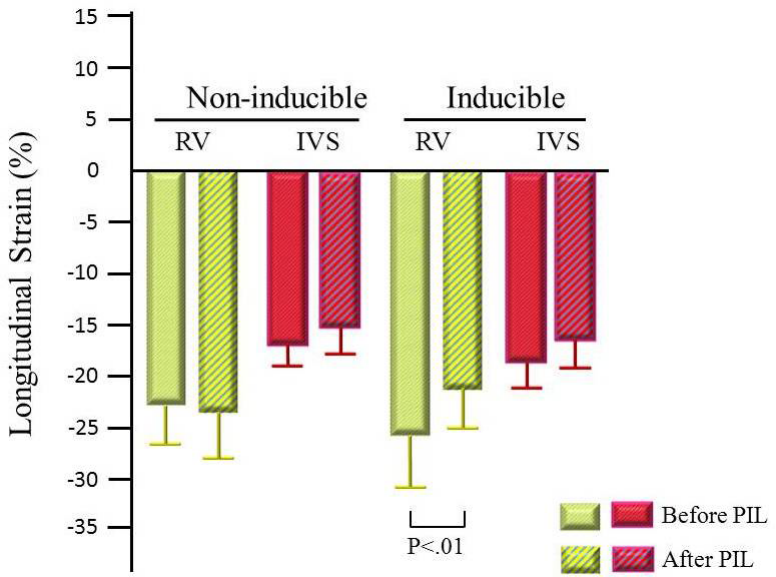
**Changes in longitudinal strain before and after pilsicainide infusion**. Longitudinal strain at the base of both the RV and IVS in the non-inducible group did not change; however, longitudinal strain at the base of the RV in the inducible group was significantly reduced after pilsicainide infusion. PIL: pilsicainide

**Figure 4:**
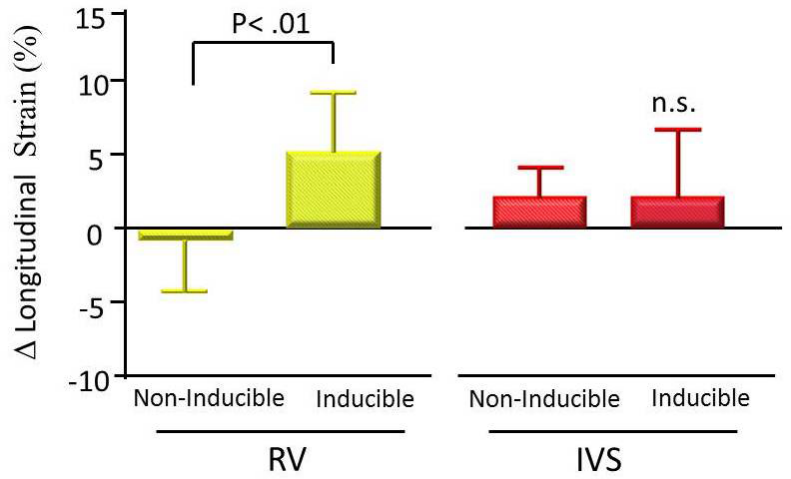
**Reduction in longitudinal strain of the RV and the IVS after pilsicainide in the inducible and non-inducible groups**. The reduction in RV longitudinal strain after pilsicainide was greater in the inducible group compared with the non-inducible group.

Longitudinal strain at the base of both RV and IVS in the non-inducible group did not change throughout the study as revealed by 2D strain imaging. However, longitudinal strain at the base of the RV in the inducible group decreased significantly after pilsicainide (-27.3 ± 5.4% vs -22.1 ± 3.6%, *P *< 0.01) (Figures 2, 3). The reduction of RV longitudinal strain after pilsicainide was greater in the inducible group compared with that in the non-inducible group (6.7 ± 2.8% vs -0.6 ± 3.7%, P < 0.01) (Figure 4). There was no difference in the reduction of IVS longitudinal strain between the non-inducible and inducible groups.

#### Intraobserver and Interobserver Reproducibility

For intraobserver reproducibility, Pearson's correlations for longitudinal strain at the base of the RV free wall and the IVS were r = 0.97 (P < 0.001) and r = 0.86 (P < 0.001), respectively. The limits of agreement for intraobserver reproducibility were -6.0% to 5.0% in the RV free wall and -3.3% to 2.8% in IVS. For interobserver reproducibility, Pearson's correlations at the base of the RV free wall and the IVS were r = 0.86 (P < 0.001) and r = 0.81 (P < 0.001), respectively. The limits of agreement for intraobserver reproducibility were -3.2% to 8.7% in the RV free wall and -2.1% to 3.3% in the IVS.

## Discussion

In this study, we observed the reduction of longitudinal strain of basal segment of RV by 2D speckle tracking techniques in patients with Brugada-type ECG and a positive response to pilsicainide.

Brugada syndrome is characterized as ST-segment elevation from a depressed RV epicardial action potential dome or a RV subepicardial action potential shortening [11,12]. Thus, Brugada syndrome has been described as "RV cardiomyopathy". Some reports have revealed wall motion abnormalities of the right ventricle in patients with Brugada syndrome using cardiac CT [3,7] and MRI [13]. Modulation of wall motion by a sodium channel blocker associated with ST-T elevation has also been found using electron beam computed tomography [3]; however, no echocardiographic studies demonstrated structural or functional abnormalities in this syndrome.

### RV longitudinal strain using 2D speckle tracking

Although previous studies reported a delay in the timing of the onset of RV contraction using tissue Doppler imaging in type 1 Brugada patients, regional RV or LV function in such situations was not fully evaluated [6,14]. In these previous studies, regional ventricular function was evaluated using the tissue Doppler method; however, impairment of RV tissue systolic velocity on the development of ST elevation was not detected. We measured longitudinal peak systolic strain as an index of regional myocardial function using the 2D strain technique and demonstrated depressed RV longitudinal strain. This technique is a new method for quantifying regional myocardial deformation based on high spatial resolution speckle tracking without angle dependency [15-17], and is not affected by translation or tethering from the surrounding tissue [17-19]. Although the high variability has been concerned for 2D strain analysis especially for right ventricle, the evaluation of right as well as left ventricular functions using this technique has been accepted [20,21]. In the present study, we could also confirm acceptable reproducibility for measuring myocardial strain by this method. Previous studies have found that electrical heterogeneity at the epicardium of the RV is related to the genesis of Brugada syndrome [11,12]. The augmentation of ST-segment elevation by class IC anti-arrhythmic drugs has been reported [2,4,22], and a possible mechanism for this augmentation in patients with Brugada syndrome has been proposed [4,11]. The loss of the RV epicardial, but not endocardial, action potential dome, which is facilitated by strong sodium channel blockers creates a transmural voltage gradient that manifests as an ST-segment elevation. This change of the action potential dome by pilsicainide may cause attenuation of myocardial contractile function and reduction of RV longitudinal strain.

### TEI index and pilsicainide challenge

Both the RV and LV TEI indices deteriorated in the inducible group; however, these indices did not deteriorate in the non-inducible group. Although previous study showed that the RV ejection time shortened as the Brugada ECG pattern emerged after flecainide infusion [6], we did not find a significant change in ejection time in either the non-inducible or inducible group. Because pilsicainide has a less prolonged QRS duration than flecainide [23], differences in electrophysiological effects may contribute to the response of the ejection time. In our study, despite no change in the ejection time, prolongation of the PR interval, especially in the inducible group, was observed after pilsicainide. Because the PR interval affected the ICT, the prolonged PR caused prolongation of ICT and resulted in deterioration of the TEI index. In addition to a negative inotropic effect of pilsicainide, reduced RV function might affect LV function through RV and LV interaction. This might have caused the deterioration of the LV- TEI index in the inducible group.

### Study Limitations

A previous study reported that sodium channel blocker administration resulted in ST-segment elevation and RBBB in a patient with an SCN5A mutation [4]. We did not perform genetic tests on the patients; therefore, the relationship between pilsicainide challenge and genetic tests is not known. Predicting the risk of cardiac events in patients with Brugada-type ECG is important. In the present study, since the number of study group was small and the patients who showed positive response were limited, we could not demonstrate the relation between RV strain after pilsicainide infusion and incidence of fatal arrhythmias. Although further investigation should be necessary, this presented results may help risk stratification for patients with Brugada-like ECG, and may facilitate to perform electrophysiologic study and to decide the indication of implantable cardioverter defibrillator.

## Conclusions

Although conventional echocardiography failed to detect structural abnormalities in patients with Brugada-type ECG and coved type ST elevation induced by a sodium channel blocker, temporal and spatial analysis by TEI index and 2D strain imaging revealed the deterioration of global ventricular function associated with the manifestation of conduction disturbance and the reduction of RV regional function.

## List of abbreviations

RV: right ventricle/ventricular; LV: left ventricle/ventricular; 2D: two-dimensional; TEI index: myocardial performance index; TDI: tissue Doppler imaging; ICT: isovolumic contraction time.

## Competing interests

The authors declare that they have no competing interests.

## Authors' contributions

KM conceived the study, participated in the study, performed echocardiographic studies and drafted the manuscript. TU participated in the study design of study. TT and YN participated in the study and performed echocardiographic studies. YW performed echocardiographic and statistical analyses. MM participated in echocardiographic studies and in the study design. All authors have read and approved the final manuscript.
